# Genetic Causal Relationship Between Systemic Lupus Erythematosus and Malignant Tumors of the Female Reproductive System: A GWAS Analysis in European Populations

**DOI:** 10.1155/humu/7447886

**Published:** 2025-05-15

**Authors:** Jianbin Li, Zhuo Tang, Lei Zhang, Ning Tan, Wei Liu

**Affiliations:** ^1^Department of Rheumatism and Immunity, First Teaching Hospital of Tianjin University of Traditional Chinese Medicine, Tianjin, China; ^2^Department of Rheumatism and Immunity, National Clinical Research Center for Chinese Medicine Acupuncture and Moxibustion, Tianjin, China; ^3^Department of Rheumatism and Immunity, Tianjin Academy of Traditional Chinese Medicine, Tianjin, China; ^4^Department of Rheumatism and Immunity, Shenzhen Nanshan People's Hospital, Shenzhen, China

**Keywords:** cross-trait analysis, female reproductive system malignancies, genome-wide association study, systemic lupus erythematosus

## Abstract

**Background:** Systemic lupus erythematosus (SLE) is a multisystem autoimmune disease that primarily affects women of reproductive age. Existing studies have demonstrated complex associations between SLE and various diseases, but its genetic relationship with malignant tumors of the female reproductive system has not been fully elucidated. This study is aimed at exploring the potential genetic associations and shared molecular basis between SLE and female reproductive system malignancies using genome-wide association studies (GWASs) and cross-trait analysis.

**Methods:** We selected genetic variants significantly associated with SLE (*p* < 5 × 10^−8^) from large-scale GWAS databases as genetic instruments and applied various statistical methods to analyze the associations between SLE and cervical cancer, endometrial cancer, ovarian cancer, vulvar cancer, vaginal cancer, and uterine cancer. The primary analysis was conducted using inverse variance weighting (IVW), supplemented by Egger regression, weighted median, and weighted mode methods. To control for potential confounders, we performed multivariable analysis while including BMI, estradiol, and CRP as covariates. Additionally, cross-trait analysis using the association analysis based on subset (ASSET) method was employed to identify shared genetic variants and their effect directions between SLE and uterine cancer.

**Results:** Genetic association analysis showed a significant negative association between SLE and endometrial cancer (OR = 0.972, 95% CI [0.946–0.998], *p* = 0.038), suggesting that SLE may be associated with a reduced risk of endometrial cancer. For uterine cancer, the weighted median method also indicated a marginally significant negative association (OR = 0.955, 95% CI [0.912–1.000], *p* = 0.049). Multivariable analysis further confirmed that the protective association between SLE and endometrial cancer remained significant after controlling for BMI, estradiol, and CRP (OR = 0.96, 95% CI [0.93–0.99], *p* = 0.014). However, no significant association was observed between SLE and cervical cancer, ovarian cancer, vulvar cancer, or vaginal cancer. Cross-trait analysis identified 193 shared genetic variants between SLE and endometrial cancer and 71 shared variants between SLE and uterine cancer, with rs2442719 and rs3131004 showing consistent effect directions in both comparisons.

**Conclusion:** This study provides genetic epidemiological evidence suggesting that SLE may have a protective effect against endometrial and uterine cancers and identifies potential shared genetic bases. These findings offer new insights into the relationship between SLE and gynecological tumors and may provide references for the prevention and treatment of related diseases.

## 1. Introduction

Systemic lupus erythematosus (SLE) is a chronic autoimmune disease characterized by the abnormal activation of the immune system and multiorgan involvement. The global prevalence is approximately 13–7713.5/100,000, with significantly higher rates in females compared to males, especially during the reproductive age, where the female-to-male ratio is as high as 9:1 [1]. SLE patients face significant health challenges not only from the disease itself but also from various comorbidities and complications, including cardiovascular diseases, infections, and malignancies [2]. This marked gender difference and variation in cancer risk have sparked ongoing research into the potential links between SLE and various cancers.

The role of the immune system in tumor initiation and progression has gained increasing attention, and the association between autoimmunity and cancer has become a major focus of research over the past decade. Numerous epidemiological studies have shown that the cancer risk in SLE patients differs significantly from that of the general population, with this association exhibiting a notable dichotomy. SLE patients have an increased risk of specific cancer types, such as non-Hodgkin lymphoma (NHL) and leukemia, as well as lung cancer, thyroid cancer, and vulvar cancer [3]; however, studies have also suggested that SLE may reduce the risk of hormone-sensitive cancers, such as breast cancer, endometrial cancer, and ovarian cancer [4]. Several hypotheses have been proposed regarding the potential mechanisms underlying the relationship between SLE and cancer, including drug exposure, interactions between drugs and viral exposure, clinical features (e.g., comorbid Sjogren's syndrome), lifestyle factors, and inherent immune system abnormalities [5]. Additionally, altered viral clearance, particularly for viruses closely related to gynecological cancers, such as human papillomavirus (HPV), may be an important factor influencing the risk of specific cancers in SLE patients [6].

Regarding malignant tumors of the female reproductive system, existing research presents a complex and sometimes contradictory picture. Although a meta-analysis showed a significantly lower risk of endometrial cancer (standardized incidence ratio (SIR) 0.72, 95% confidence interval (CI) 0.56–0.91) and ovarian cancer (SIR 0.69, 95% CI 0.50–0.90) in SLE patients [4], these observational studies are limited by the inability to fully control for confounding factors such as hormone exposure, drug treatment (especially long-term use of antimalarials and immunosuppressants), and more frequent medical follow-ups in SLE patients [7]. Furthermore, epidemiological studies typically fail to reveal the molecular mechanisms underlying these associations, particularly the potential shared pathways at the genetic level [8]. Given that both SLE and female reproductive system malignancies have distinct gender preferences and potential hormone-regulated features, exploring the genetic causal relationship between these two diseases is of significant importance for understanding their pathogenesis.

In recent years, with the rapid development of genomic research, genome-wide association studies (GWASs) have identified numerous genetic variants associated with SLE [9], providing a solid foundation for exploring the genetic links between SLE and other diseases. An interesting hypothesis is that specific genetic factors may predispose individuals to SLE while simultaneously offering protection against certain cancers, such as breast cancer [10]. Cross-trait analysis, as an innovative statistical method, integrates GWAS data from different traits and can effectively identify shared genetic bases between diseases, offering new perspectives on the molecular connections between different diseases [11]. Previous studies have attempted to explore the genetic associations between SLE and some malignancies [12], but systematic evaluations of the genetic relationship between SLE and female reproductive system malignancies remain limited.

This study, based on large-scale European population GWAS data, uses the association analysis based on subset (ASSET) method for cross-trait analysis, aiming to explore the genetic causal relationship between SLE and female reproductive system malignancies. We systematically analyzed shared genetic variants and their effect directions between these diseases, hoping to provide new insights into the biological links between SLE and female reproductive system malignancies and offer a theoretical basis for risk prediction and prevention strategies for related diseases.

## 2. Methods

### 2.1. Study Design

**Figure 1:**
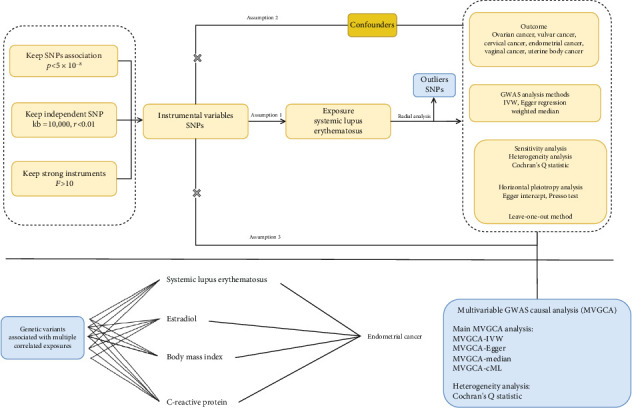
The process of study and genetic causal inference analysis. This figure presents a comprehensive overview of the study workflow, highlighting the sequential steps from data acquisition to analysis. It begins with the identification of exposure and outcome data sources, followed by the criteria used for SNP extraction. The genetic causal inference analysis is based on three core assumptions: (1) SNP-exposure association (genetic variants robustly correlated with exposure), (2) independence assumption (instrumental variables not associated with confounders), and (3) direct effect exclusion (instrumental variables affected outcome only through the risk factor). All analyses were performed using specialized packages in R software (Version 4.3.1), including tools for two-sample analysis (Version 0.5.11), radial analysis (Version 1.1), and other related packages to assess heterogeneity and pleiotropy and conduct multivariable analyses. The workflow diagram was created using Adobe Illustrator (2024 version).

This study utilized genetic summary data from the FinnGen and GWAS Catalog databases to evaluate the genetic associations between SLE and the risk of malignant tumors of the female reproductive system using a GWAS-based genetic association analysis approach. Genetic variants were selected according to strict criteria based on genetic epidemiology standards: (1) variants significantly associated with SLE (*p* < 5 × 10^−8^, linkage disequilibrium (LD) threshold *r*^2^ < 0.001, and genetic distance > 10,000 kb), (2) independent of potential confounders related to both SLE and female reproductive system malignancies, and (3) excluding pleiotropic variants influencing female reproductive system malignancies through pathways other than SLE. The primary analysis employed inverse variance weighting (IVW) to estimate the association effects. IVW integrates the inverse of the variance of genetic variants as weights in regression fitting, offering optimal statistical power under the assumption of no horizontal pleiotropy. Additionally, Egger regression and weighted median methods were used for sensitivity analysis, with the former correcting for directional pleiotropy bias and the latter being robust to invalid genetic variants. To ensure the reliability of the results, several strategies were implemented to control potential biases: (1) Cochran's *Q* test was used to assess genetic variant heterogeneity (with a *p* value > 0.05 suggesting the retention of a fixed effects IVW model), (2) Egger intercept test and global tests were used to identify horizontal pleiotropy (*p* > 0.05 considered no significant pleiotropy), (3) leave-one-out (LOO) sensitivity analysis was performed to exclude SNP-driven effects, and (4) radial IVW plots were used to identify and assess genetic variants that may overly influence the overall effect estimate. To control for the potential confounding factors affecting the association between SLE and endometrial cancer, we conducted a multivariable genetic association analysis, incorporating BMI, estradiol, and CRP as covariates. The BMI data were obtained from the FinnGen database (https://storage.googleapis.com/finngen-public-data-r12/summary_stats/release/finngen_R12_BMI_IRN.gz), which included phenotype, genotype, and clinical information from 362,327 individuals of European ancestry. Estradiol data were sourced from the GWAS Catalog (https://www.ebi.ac.uk/gwas/), including phenotype, genotype, and clinical data from 206,927 individuals of European ancestry. CRP data were obtained from a dataset including phenotype, genotype, and clinical information from 5368 individuals of European ancestry. The multivariable analysis employed four complementary statistical methods: multivariable IVW, multivariable Egger regression, median method, and conditional maximum likelihood (cML) method [13–15]. To assess the validity of the genetic instruments, Cochran's *Q* statistic was used to test for residual heterogeneity in the model. All variants were rigorously screened, and SNPs with extremely small standard errors (SEs) were excluded. The final analysis included 333 valid SNPs (308 related to SLE). The results from the multivariable analysis were compared with those from the univariate analysis to assess the robustness of the association between SLE and endometrial cancer before and after adjusting for confounders. The overall study design flow is shown in Figure 1.

### 2.2. Data Source and Instrumental Variable (IV) Selection

The data for SLE were obtained from the IEU Open GWAS (https://gwas.mrcieu.ac.uk), including phenotype, genotype, and clinical information from 12,653 individuals of European ancestry. Ovarian cancer data came from the GWAS Catalog (https://www.ebi.ac.uk/gwas/), which includes phenotype, genotype, and clinical information from 247,508 individuals of European ancestry. Cervical cancer data were sourced from the GWAS Catalog (https://www.ebi.ac.uk/gwas/), including phenotype, genotype, and clinical information from 383,561 individuals of European ancestry. Endometrial cancer data were obtained from the GWAS Catalog (https://www.ebi.ac.uk/gwas/), including phenotype, genotype, and clinical information from 247,540 individuals of European ancestry. Vulvar cancer data were retrieved from the FinnGen database (https://storage.googleapis.com/finngen-public-data-r12/summary_stats/release/finngen_R12_CD2_INSITU_VULVA_EXALLC.gz), with data from 222,185 individuals of European ancestry. Vaginal cancer data were obtained from the FinnGen database (https://storage.googleapis.com/finngen-public-data-r12/summary_stats/release/finngen_R12_C3_VAGINA_EXALLC.gz), including phenotype, genotype, and clinical information from 222,180 individuals of European ancestry. Uterine cancer data were sourced from the FinnGen database (https://storage.googleapis.com/finngen-public-data-r12/summary_stats/release/finngen_R12_C3_CORPUS_UTERI_EXALLC.gz), including phenotype, genotype, and clinical information from 225,568 individuals of European ancestry. The IVs for this study were selected based on the following criteria: a genome-wide significance threshold was set at *p* < 5 × 10^−8^, and a LD threshold of *r*^2^ < 0.001 (kb = 10,000) was used to ensure effective LD [16]. In addition, to minimize weak instrument bias, the *F*-statistic was calculated for each SNP. IVs were selected using the *F*-statistic method: *F*‐statistic = *R*^2^ × (*N* − 2)/(1 − *R*^2^)。*R* = 2 × EAF × (1 − EAF) × beta^2^ [17, 18]. SNPs with an *F*-value < 10 were considered weak and excluded from further analysis. For comprehensive details on the genetic instruments selected for SLE and ovarian cancer, refer to Table 1. Additionally, given the potential differences between proxy SNPs and the original SNPs, proxy SNPs were excluded from the analysis [19, 20]. Finally, radial MR methods were used to remove outliers, ensuring the reliability of causal inference [21].

### 2.3. Cross-Trait Analysis

To further evaluate the shared genetic variants between SLE and endometrial cancer and uterine cancer, we performed a cross-trait analysis using the ASSET method [11]. The ASSET method combines the GWAS results from different traits (SLE, endometrial cancer, and uterine cancer) through a weighted merging process, which allows the identification of shared genetic variants across traits. In this analysis, we used the h.traits function from the ASSET package in R to calculate the effect sizes and SEs of each SNP and assessed whether there were shared genetic variants between SLE and endometrial cancer, as well as SLE and uterine cancer. This method accounts for sample overlap and heterogeneity in effect sizes, effectively identifying SNPs with consistent effect directions across traits. Our analysis flow is as follows: First, we extracted SNP information, effect sizes (beta), and SEs from each GWAS study. We then used the following parameters to call the h.traits function: two-tailed test (side = 2), meta-analysis (meta = TRUE), and *p* value calculation using the DLM method. For each disease combination, we recorded the number of cases (ncase) and controls (ncntl). For the identified shared SNPs, we set the statistical significance threshold at *p* < 0.05 and selected variants that reached significance in both diseases with consistent effect directions. For the key SNPs identified, we used the UCSC Genome Browser (https://genome.ucsc.edu/cgi-bin/hgTracks) to query their positional information for subsequent functional annotation analysis [22]. For the key SNPs identified in the cross-trait analysis, we further conducted a more detailed meta-analysis using the metagen function from the meta package in R, while assessing both fixed effects and random effects models. For each SNP, we analyzed its effect consistency and heterogeneity between SLE and endometrial cancer, as well as between SLE and uterine cancer. We used Cochran's *Q* test and the *I*^2^ statistic to assess the degree of heterogeneity, with *I*^2^ indicating the proportion of variance in effect sizes due to study heterogeneity. Due to significant heterogeneity observed (*I*^2^ > 85%, *p* < 0.01), we employed a random effects model based on restricted maximum likelihood (REML) estimation to estimate the effects, which provides a more conservative but potentially more accurate assessment of effect differences across diseases [23].

### 2.4. Statistical Analyses

We conducted sensitivity analyses to assess potential biases. First, we used Cochran's *Q* statistic to evaluate the heterogeneity between genetic variants. A *p* value > 0.05 indicates no significant heterogeneity. When significant heterogeneity (*p* < 0.05) was detected, we applied a multiplicative random effects model with IVW for robust estimation. To enhance the reliability of causal inference, we employed the PRESSO (Pleiotropy RESidual Sum and Outlier) method to identify and remove potential outlier SNPs that could be caused by horizontal pleiotropy. Specifically, we performed a global PRESSO test on all SNPs to identify potential outliers. After each outlier SNP was removed, we repeated the process until the global test *p* value was no longer significant (*p* > 0.05), ensuring that all outlier SNPs were fully excluded. Subsequently, we conducted a LOO analysis by sequentially removing each SNP and observing changes in effect estimates to evaluate the influence of each individual SNP on the overall causal effect estimation. Additionally, we used Egger regression to assess horizontal pleiotropy by testing the significance of the Egger intercept. If the *p* value for the Egger intercept was > 0.05, it indicated that no significant horizontal pleiotropy was present under the Egger model, which aligns with the core assumption of genetic instrument variable analysis.

## 3. Results

### 3.1. Selection of IVs

We selected genetic variants (SNPs) significantly associated with SLE (*p* < 5 × 10^−8^) from the genome-wide data. To ensure that the selected SNPs were sufficiently independent, we set the LD parameters to *r*^2^ > 0.001 and kb = 10,000 and excluded any SNPs that did not meet these criteria. As a result, 42 independent SNPs were identified.

Next, we extracted SNPs associated with cervical cancer, endometrial cancer, ovarian cancer, vulvar cancer, vaginal cancer, and uterine cancer from publicly available databases. We identified 41, 39, 41, 39, 39, and 39 SNPs, respectively, from these cancer types that satisfied the following criteria: Hypotheses 1, 2, and 3 (see Supporting Information 1: Table S1). All selected IVs had *F*-statistic values greater than 10, indicating that these IVs had sufficient strength in this GWAS analysis, thereby confirming the reliability of the results.

### 3.2. Causal Effect of SLE on Malignant Tumors of the Female Reproductive System

**Figure 2:**
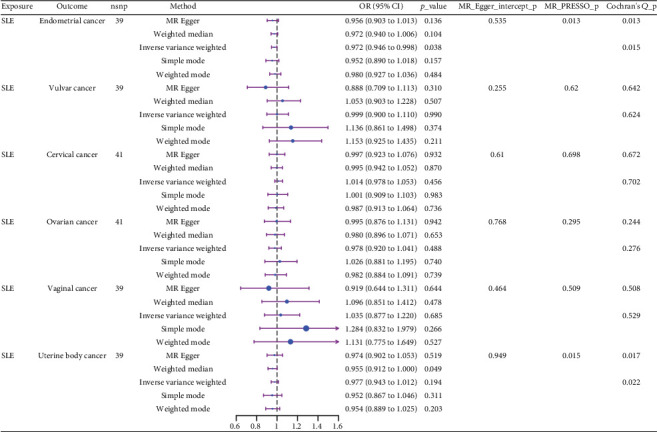
Causal effects of systemic lupus erythematosus on female reproductive system malignancies in GWAS analysis.

**Figure 3:**
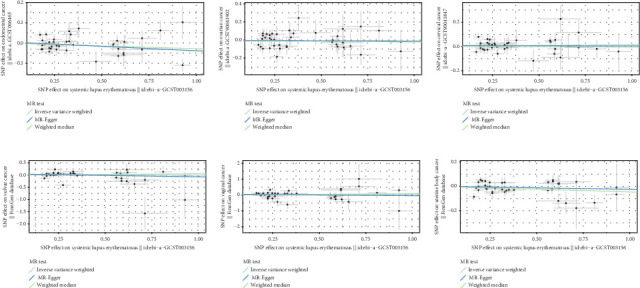
(a–f) Scatter plot of the causal effect of SLE on malignant tumors of the female reproductive system.

We explored the causal relationship between SLE and female reproductive system malignancies through GWAS analysis, revealing several noteworthy findings. Using IVW as the primary analytical method, we found a significant causal relationship between SLE and endometrial cancer (odds ratio (OR) = 0.972, 95% CI [0.946–0.998], *p* = 0.038). This suggests that SLE may have a protective effect on endometrial cancer. For vulvar cancer, the analysis showed no significant causal relationship with SLE (OR = 0.999, 95% CI [0.900–1.110], *p* = 0.990). Similarly, no significant causal relationship was found between SLE and cervical cancer (OR = 1.014, 95% CI [0.978–1.053], *p* = 0.456), ovarian cancer (OR = 0.978, 95% CI [0.920–1.041], *p* = 0.488), vaginal cancer (OR = 1.035, 95% CI [0.877–1.220], *p* = 0.685), or uterine cancer (OR = 0.977, 95% CI [0.943–1.012], *p* = 0.194). The results from other GWAS methods, including Egger regression, weighted median, and weighted mode, were generally consistent with our primary IVW analysis. For uterine cancer, the weighted median method showed a marginally significant result (OR = 0.955, 95% CI [0.912–1.000], *p* = 0.049). Heterogeneity tests, including Cochran's *Q* test (all *p* > 0.244) and MR-PRESSO test (all *p* > 0.015), did not show significant heterogeneity in most analyses. For endometrial cancer and uterine cancer, the MR-PRESSO test indicated potential pleiotropy (*p* = 0.013 and *p* = 0.017, respectively), but the corrected estimates remained consistent with the primary analysis results. Overall, our comprehensive GWAS analysis of multiple female reproductive system malignancies suggests a significant negative causal relationship between SLE and endometrial cancer in the European population, indicating that SLE may have a protective effect on endometrial cancer. Additionally, we observed a potential marginal protective effect of SLE on uterine cancer (OR = 0.955, 95% CI [0.912–1.000], *p* = 0.049, weighted median method), a finding that warrants further investigation. For other female reproductive system malignancies (vulvar cancer, cervical cancer, ovarian cancer, and vaginal cancer), no significant causal relationships with SLE were found. The results from the five commonly used GWAS analysis methods are shown in Figures 2 and 3.

### 3.3. Negative Correlation Between SLE and Endometrial Cancer and Uterine Cancer

**Figure 4:**
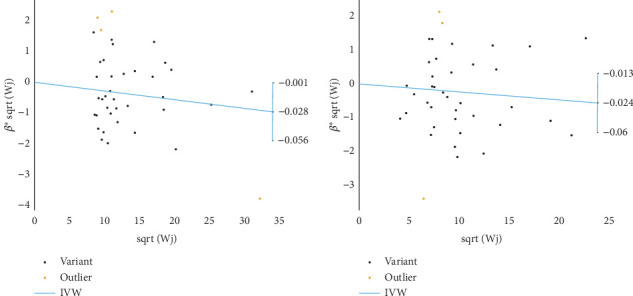
Inverse variance weighting (IVW) analysis scatter plot of systemic lupus erythematosus and endometrial cancer and uterine cancer. (a) Causal relationship analysis between SLE and endometrial cancer, showing a negative association (slope = −0.028). (b) Causal relationship analysis between SLE and uterine cancer, also presenting a negative association (slope = −0.024). Black points represent the effect estimates of each SNP as an independent instrumental variable, with the *x*-axis indicating the weight of each SNP (√Wi) and the *y*-axis showing the standardized effect size (*β*i/sei). Yellow points mark outlier SNPs (those outside the 95% confidence interval). The blue line represents the IVW regression line and its 95% confidence interval. In both plots, most SNPs are within the confidence interval, supporting the conclusion that SLE may have a protective effect against these two types of cancer.

Based on GWAS analysis, the IVW method revealed a statistically significant negative causal relationship between SLE and both endometrial cancer and uterine cancer, suggesting that SLE may have a protective effect against endometrial cancer. Further examination of the IVW radial plot shows that the majority of genetic IVs fall within the CI, although a few outlier points (marked in yellow) are present. Overall, the data distribution is reasonable. The negative slopes of the lines (−0.028 for endometrial cancer and −0.024 for uterine cancer) further confirm the negative association between SLE and these two cancers (Figure 4).

### 3.4. Sensitivity Analysis

**Figure 5:**
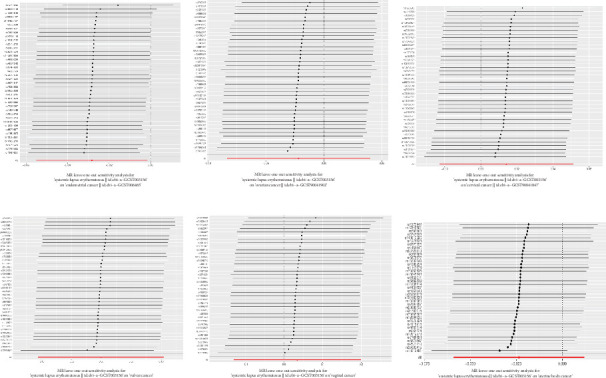
Leave-one-out sensitivity analysis of systemic lupus erythematosus and female reproductive system malignancies. The black points represent the log odds ratios (OR) for the effect of each standard deviation (SD) increase in SLE on endometrial cancer, vulvar cancer, cervical cancer, ovarian cancer, vaginal cancer, and uterine cancer, as estimated using each SNP as an individual instrumental variable. The red points and red horizontal line represent the combined causal estimates using all SNPs as the overall instrumental variables with the inverse variance weighting (IVW) method, along with their 95% confidence intervals. The six subplots (a–f) display the leave-one-out sensitivity analysis results for SLE on the six different types of female reproductive system malignancies, testing the stability of the estimates after removing any individual SNP. The vertical dashed line indicates the null effect value (OR = 1). The distribution of points is relatively concentrated, with most estimates aligning with the combined estimate, particularly in the analysis of endometrial cancer (a) and uterine cancer (f), which supports the robustness of the causal relationship estimates between SLE and these cancers.

**Figure 6:**
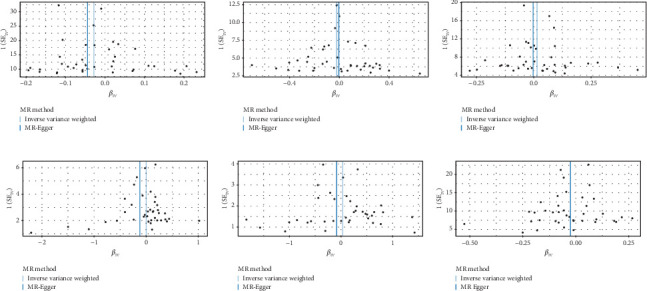
(a–f) The funnel plot showed the instrumental variables with relatively symmetrical divisions. Funnel plot of two-sample GWAS analysis results. The blue line represents the inverse variance weighting (IVW) estimate, and the dark blue line represents the Egger estimate.

The GWAS analysis evaluating the relationship between SLE and female reproductive system malignancies using the IVW method showed heterogeneity test results as follows for different cancers: endometrial cancer (Cochran's *Q*_*p* = 0.013), cervical cancer (Cochran's *Q*_*p* = 0.672), ovarian cancer (Cochran's *Q*_*p* = 0.244), vulvar cancer (Cochran's *Q*_*p* = 0.642), vaginal cancer (Cochran's *Q*_*p* = 0.508), and uterine cancer (Cochran's *Q*_*p* = 0.017). Although the *p* values for endometrial cancer and uterine cancer were < 0.05, indicating potential heterogeneity, no significant heterogeneity was observed for the other cancer types. The MR-Egger intercept method for evaluating horizontal pleiotropy showed that all pleiotropy *p* values (endometrial cancer = 0.535, cervical cancer = 0.61, ovarian cancer = 0.768, vulvar cancer = 0.255, vaginal cancer = 0.464, and uterine cancer = 0.949) were > 0.05, suggesting that there was no significant horizontal pleiotropy in the genetic IVs. The PRESSO test indicated the possibility of outliers for endometrial cancer (*p* = 0.013) and uterine cancer (*p* = 0.015), but the corrected estimates were consistent with the original analysis results. LOO sensitivity analysis further confirmed (Figure 5) that the GWAS estimates for each cancer remained stable even after excluding any individual SNP, suggesting that the results were not significantly influenced by any individual SNP. The funnel plot (Figure 6) showed a symmetrical distribution, supporting the robustness of the causal relationship estimates between SLE and the associated female reproductive system cancers. Overall, the combined analysis supports a robust protective causal relationship between SLE and female reproductive system malignancies.

### 3.5. Multivariable Analysis

To further control for potential confounding factors, we conducted a multivariable genetic association analysis to assess the direct effect of SLE on the risk of developing endometrial cancer, while considering potential confounders such as BMI, estradiol, and CRP (Table 2). We employed several statistical methods to ensure the robustness of the results. In the model that accounted for these multiple factors, a significant negative association between SLE and endometrial cancer risk was observed (IVW method: OR = 0.96, 95%CI = 0.93–0.99, *p* = 0.014; Egger method: OR = 0.96, 95%CI = 0.93–0.99, *p* = 0.017; conditional likelihood method: *p* = 1.91 × 10^−2^). This further supports our primary finding that SLE may have a protective effect against endometrial cancer. Importantly, this association remained significant after controlling for BMI, estradiol, and CRP, indicating that the negative association between SLE and endometrial cancer is independent of these factors. Additionally, BMI showed a significant positive association with endometrial cancer risk (IVW method: OR = 1.53, 95%CI = 1.29–1.82, *p* < 0.001; Egger method: OR = 2.19, 95%CI = 1.29–3.72, *p* = 0.004; conditional likelihood method: *p* = 3.04 × 10^−7^), which is consistent with previous studies. However, after controlling for other factors, neither estradiol (IVW method: OR = 0.55, 95%CI = 0.04–6.94, *p* = 0.646) nor CRP (IVW method: OR = 0.99, 95%CI = 0.90–1.09, *p* = 0.814) showed statistically significant associations with endometrial cancer risk in the multivariable model, despite both showing significant associations in univariate conditional likelihood analysis (*p* values were 6.30 × 10^−4^ and 6.37 × 10^−4^, respectively).

Table 2 provides an overview of the multivariable analysis results, primarily examining the associations between various exposure factors (BMI, estradiol, SLE, and CRP) and the risk of endometrial cancer. The results include the ORs, 95% CIs, and *p* values for each factor across different statistical methods (IVW, Egger, and conditional likelihood). The analysis assesses the direct effect of SLE on endometrial cancer risk while controlling for the potential confounding effects of BMI, estradiol, and CRP. Additionally, the table summarizes the sensitivity analysis findings, including results from the LOO analysis and heterogeneity tests.

### 3.6. SLE and Shared Genetic Variants With Uterine Cancer

Through GWAS analyses of SLE with endometrial cancer and uterine cancer, we applied the ASSET method for cross-trait analysis and successfully identified multiple shared genetic variants. In the analysis between SLE and endometrial cancer, we identified 193 shared SNPs, all of which exhibited statistically significant associations (*p* < 0.05) in both diseases with consistent effect directions. Similarly, in the SLE and uterine cancer analysis, we identified 71 shared genetic variants, which also showed consistent effect directions and statistical significance in both diseases. Notably, rs2442719 and rs3131004 were the only SNPs identified in both comparisons, and their cross-appearance highlights their unique potential as shared genetic bases between SLE and different types of uterine cancer. All identified shared genetic variants are detailed in Supporting Information 2: Table S2. To further validate the shared genetic association of these two SNPs, we performed a meta-analysis. Under the fixed effects model, both rs2442719 and rs3131004 exhibited significant shared genetic associations between SLE and endometrial cancer as well as uterine cancer (*p* < 0.0001), with consistent negative effect directions. However, due to observed high heterogeneity (*I*^2^ values of 96.5%, 91.0%, 94.5%, and 85.4%), we further employed a random effects model for analysis. Under the random effects model, most associations were no longer statistically significant, except for rs3131004, which remained significant in the association between SLE and uterine cancer (*β* = −0.1108, *p* = 0.0280). Despite this, all SNPs maintained consistent negative effect directions, suggesting the presence of shared biological mechanisms, although the effect sizes may vary between different diseases. Both SNPs are located in the major histocompatibility complex (MHC) region on Chromosome 6, with rs2442719 located between Positions 31352661 and 31352861 and rs3131004 located between Positions 31127417 and 31127617. The MHC region is crucial for immune function, and this finding supports the potential shared immune regulatory mechanisms between autoimmune diseases, such as SLE, and certain cancer types. Notably, rs3131004 remained significant in the random effects model, suggesting its potential role in the shared pathogenesis between SLE and uterine cancer. Detailed results of the meta-analysis, including the fixed effects and random effects models as well as heterogeneity analysis, are presented in Table 3.

## 4. Discussion

This study systematically explored the genetic causal relationship between SLE and female reproductive system malignancies using GWAS and cross-trait analysis methods, revealing several clinically significant findings. Our analysis showed a significant negative causal relationship between SLE and endometrial cancer (OR = 0.972, 95% CI [0.946–0.998]), suggesting that SLE may have a protective effect on endometrial cancer. This finding remained robust after controlling for potential confounding factors such as BMI, estradiol, and CRP (OR = 0.96, 95% CI [0.93–0.99]). Additionally, we observed a marginally significant negative association between SLE and uterine cancer using the weighted median method (OR = 0.955, 95% CI [0.912–1.000]). However, no significant genetic causal relationship was found between SLE and cervical cancer, ovarian cancer, vulvar cancer, or vaginal cancer.

Using the ASSET method, we identified 193 shared SNPs between SLE and endometrial cancer and 71 shared SNPs between SLE and uterine cancer, providing direct evidence of the genetic connection between SLE and gynecological cancers. Notably, rs2442719 and rs3131004 were the only SNPs identified in both comparisons, showing consistent effect directions. These two SNPs demonstrated significant associations with all comparisons under the fixed effects model (*p* < 0.0001), but due to observed high heterogeneity (*I*^2^ > 85%), the random effects model might provide more reliable estimates. Notably, rs3131004 remained significantly associated with SLE–uterine cancer in the random effects model (*β* = −0.1108, *p* = 0.0280), suggesting its potentially important role in this association.

The high heterogeneity observed is of significant biological importance. It may reflect the variability of the SLE–uterine cancer relationship across different molecular subtypes or clinical phenotypes and may also indicate complex gene–environment interactions [24]. Therefore, the significant result for rs3131004 under the random effects model is particularly noteworthy, potentially representing a relatively stable genetic association that is less influenced by subtype differences or environmental factors.

These findings are highly consistent with previous epidemiological research. In a meta-analysis of 47,325 SLE patients, Bernatsky et al. found that the SIR for endometrial cancer in SLE patients was 0.71 (95% CI [0.55–0.91]), suggesting a protective effect of SLE on endometrial cancer [4]. Similarly, in an international multicenter prospective cohort study, the risk of endometrial cancer was significantly reduced in 16,409 SLE patients (SIR = 0.44; 95% CI [0.23–0.77]) [3]. Our study further supports this protective association at the genetic level, controlling for common confounders. Notably, the phenomenon of reduced risk for hormone-sensitive tumors is not limited to endometrial and uterine cancer but also extends to breast cancer and potentially ovarian cancer. The consistency of this phenomenon suggests that a common mechanism may mediate the protective effect of SLE on these cancers. Bernatsky et al. showed that the protective effect of SLE on breast cancer exists across different age groups (including premenopausal and postmenopausal), indicating that this protection may not only be related to estrogen levels but may involve more complex mechanisms [4].

Among the shared genetic variants identified in our study, rs2442719 and rs3131004 are located in the MHC region, which includes several genes related to immune function. The MHC region encodes various immune-related molecules, including HLA molecules, complement proteins, and cytokines, which play a central role in immune response regulation [25]. Specifically, rs2442719 is located near the HLA-B region, while rs3131004 is located between the HLA-C and HLA-B regions. These variants may influence the expression of HLA alleles or alter the antigen presentation process, affecting T-cell recognition of tumor-associated antigens [26]. According to recent immuno-oncology research, the expression levels of HLA Class I molecules are closely related to tumor immune surveillance and immune escape [27]. We hypothesize that SLE-related MHC variants may enhance the expression of specific HLA alleles, increasing antigen presentation efficiency and thereby enhancing immune surveillance of precancerous cells in endometrial and uterine cancer. This hypothesis aligns with the recent “beneficial autoimmunity” theory in autoimmune disease patients, which suggests a protective effect against certain cancers [28]. Furthermore, MHC region variants may exert their effects by modulating the Type I interferon (IFN) pathway. Numerous studies have shown that the Type I IFN pathway is abnormally activated in SLE patients, and Type I IFNs have potent antitumor activity [29]. rs3131004 may inhibit the development of uterine cancer by enhancing the production or signaling of Type I IFNs. This aligns with recent findings that gene expression profiles related to IFNs are significantly associated with prognosis in endometrial cancer patients [30].

Several possible mechanisms explain the protective effect of SLE on endometrial cancer. One theory is that long-term use of oral immunosuppressants by SLE patients may reduce endogenous estrogen exposure, thereby lowering the risk of an estrogen-dependent endometrial cancer [31]. Although estradiol itself did not show a statistically significant association with endometrial cancer in our multivariable analysis, this may be due to sample size limitations or the fact that single-point serum estrogen measurements do not reflect long-term exposure levels. Another view suggests that SLE patients' immune surveillance may be more active against hormone-dependent cancers [4]. It is worth noting that cell death mechanisms play a crucial role in tumorigenesis and progression. For example, recent studies on cuproptosis suggest that an imbalance in copper ion levels can affect the survival and proliferation of tumor cells by inducing mitochondrial dysfunction and oxidative stress [32]. Studies have shown that SLE patients have various immune abnormalities, including increased autoantibody production, complement activation, and Type I IFN pathway activation. Notably, the activation of the Type I IFN pathway has been shown to play an important role in the suppression of various cancers. The huge progress in cancer immunotherapy in recent years has provided new perspectives for understanding the relationship between SLE and cancer. Immune checkpoint inhibitors (such as PD-1/PD-L1 and CTLA-4 inhibitors) have achieved significant clinical efficacy by enhancing T-cell antitumor activity, but they also increase the risk of autoimmune side effects [33]. This suggests that tumor immune surveillance and autoimmune diseases may share some molecular pathways. This viewpoint is also supported by studies in other types of cancer. For example, in colon adenocarcinoma, SIGLEC1, as an immune-related gene, has been confirmed to be significantly associated with immune cell infiltration in the tumor microenvironment and patient prognosis [34]. Patients with higher expression levels of SIGLEC1 often exhibit more macrophage (M0 and M2 types) infiltration and poorer survival prognosis, indicating that specific immune markers may influence the immune balance in the tumor microenvironment, thereby having a significant impact on tumor development. This may share a common immunological basis with the changes in tumor risk observed in SLE patients, reflecting the immune system's selective surveillance and regulation of different types of tumors. Future research could explore whether similar immune marker expression patterns exist in SLE patients to better understand the precise link between autoimmunity and cancer risk. Our study provides genetic support for this idea and suggests that SLE patients may naturally have enhanced tumor immune surveillance, at least for certain types of cancer such as endometrial and uterine cancer. Notably, the increased risk of some cancers in SLE patients (such as NHL) may also be related to immune system abnormalities. As Bernatsky et al. pointed out, abnormal lymphocyte proliferation (related to autoimmunity) may increase the chance of chromosomal translocations, thereby promoting the development of NHL [35]. Meanwhile, an active immune surveillance system may more effectively clear abnormal (precancerous) cells, which could explain the protective effect of SLE on certain tumors. Moreover, the medications used by SLE patients may also influence cancer risk. Studies show that long-term use of antimalarial drugs (such as hydroxychloroquine) lowers cancer risk in SLE patients [36]. Oncologists have proposed that antimalarial drugs may play a role in cancer treatment by inducing a process called autophagy [37]. Similarly, nonsteroidal anti-inflammatory drugs and aspirin have been reported to reduce the risk of certain cancers [38]. Another important consideration is altered viral clearance abilities. HPV is closely related to vulvar and cervical cancers, and SLE patients may have altered viral clearance, which could explain the differences in the risk of some gynecological cancers [39]. For younger SLE patients, HPV vaccination before sexual activity may be an effective preventive strategy.

Compared to previous studies, our GWAS analysis did not confirm a significant association between SLE and cervical cancer or ovarian cancer, which differs from some epidemiological findings. For instance, some observational studies suggest an increased risk of cervical cancer in SLE patients, possibly due to impaired HPV clearance [39]. Regarding ovarian cancer, most studies suggest a reduced risk in SLE patients, although some reports from Asian and European populations indicate an increased risk [40, 41]. These differences may reflect the varying roles of genetic and environmental factors in disease occurrence. Our study focused on genetic causal relationships, while observational studies reflect the combined effects of genetic and environmental factors. For example, cervical cancer is primarily associated with HPV infection, and SLE patients' use of immunosuppressive drugs may increase the risk of HPV infection, an environmental factor that cannot be captured in GWAS analysis. Additionally, genetic background differences across populations may lead to inconsistencies in research findings. Our analysis is mainly based on GWAS data from the European population, while SLE clinical phenotypes and genetic backgrounds vary significantly across different races [42]. Notably, our study confirmed the negative correlation between SLE and endometrial and uterine cancers from a genetic perspective, which is consistent with most epidemiological studies and suggests that this association is likely driven primarily by genetic factors and is relatively unaffected by environmental factors. This consistency between genetic and epidemiological evidence strengthens the reliability of our conclusion and provides a more comprehensive view of the relationship between SLE and cancer.

Our study systematically revealed the genetic relationship between SLE and endometrial and uterine cancers from a genetic perspective, providing new evidence for understanding the biological link between these diseases. These findings have important implications for the clinical management of SLE patients and translational medical research. From a clinical management perspective, our results can directly guide cancer risk assessment and screening strategies for SLE patients. Based on our findings, physicians can more accurately assess the risk of endometrial and uterine cancers in SLE patients, avoiding unnecessary overscreening while strengthening surveillance for high-risk cancers such as NHL. For example, SLE patients with specific HLA genotypes or rs3131004 risk alleles may require adjustments in traditional cancer screening programs. From a translational medical perspective, key genetic variants such as rs2442719 and rs3131004 provide candidate targets for developing new biomarkers and therapeutic targets. We could develop genetic risk scoring systems based on these SNPs to provide personalized cancer risk predictions for SLE patients. Additionally, by investigating the molecular pathways related to these SNPs, we may discover new drug targets that are applicable to both SLE and gynecological cancers. For example, targeting specific HLA molecules or the Type I IFN pathway might simultaneously modulate autoimmunity and tumor immune surveillance [43]. More broadly, our research provides genetic support for the concept of “beneficial autoimmunity,” opening up new strategies for tumor immunotherapy. By mimicking the beneficial immune surveillance mechanisms in autoimmune diseases like SLE while avoiding harmful autoimmune reactions, more effective and less toxic immunotherapy options may be developed [44]. For example, selectively activating the molecular pathways related to rs3131004 may enhance tumor immune surveillance without triggering significant autoimmunity. Additionally, for SLE patients, our findings highlight the importance of HPV vaccination and cervical cancer screening. While SLE may have a protective effect on endometrial and uterine cancers, environmental factors (such as HPV infection) may counteract the genetic protective effect on cervical cancer. Research shows that SLE patients may have lower screening rates than recommended [45], making patient education and healthcare system coordination crucial for improving screening compliance.

Our study also has several limitations. First, our analysis is mainly based on GWAS data from the European population, and the results may not fully apply to other racial populations. Given that SLE has significantly different incidence rates and clinical phenotypes across races, future studies should validate our findings in multiracial populations. Second, although GWAS analysis can identify genetic variants associated with disease, these variants typically explain only a small fraction of phenotypic variation. Environmental factors, epigenetic modifications, and gene–environment interactions also play important roles in disease development but are difficult to capture in GWAS analysis. Future studies should integrate multiomics data, including epigenomics, transcriptomics, and proteomics, to provide a more comprehensive understanding of disease mechanisms. Third, the high heterogeneity (*I*^2^ > 85%) observed in our study suggests that the SLE–uterine cancer association may vary across different populations or disease subtypes. This heterogeneity may reflect complex gene–environment interactions or disease subtype differences, but our current analysis could not explore these factors in detail. Future studies should consider more refined subtype classifications of SLE and uterine cancer to reduce heterogeneity and reveal more specific association patterns. Fourth, we did not distinguish between different subtypes of endometrial cancer (e.g., Type I and Type II) and ovarian cancer (e.g., serous, mucinous, and clear cell carcinoma), which have significant molecular and risk factor differences. Future research should consider these subtype differences, which may reveal more specific genetic association patterns. Finally, while we identified several shared SNPs, the specific mechanisms by which these variants influence disease development remain poorly understood. Functional studies, such as gene knockout/knock-in experiments, expression quantitative trait locus (eQTL) analyses, and single-cell sequencing, are crucial for elucidating the biological significance of these SNPs.

Based on the findings of this study, we propose several future research directions that warrant further exploration. First, functional validation studies should be conducted to explore the molecular mechanisms of key SNPs such as rs2442719 and rs3131004 in the shared pathogenesis of SLE and uterine cancer using CRISPR-Cas9 gene editing technology in cell and animal models. Second, the study should be expanded to multiracial populations, including Asian, African, and Latin American populations, to validate whether our findings in the European population are generalizable. At the same time, integrating genomic, epigenomic, transcriptomic, and proteomic data to construct molecular networks between SLE and uterine cancer could provide a more comprehensive understanding. Additionally, large prospective cohort studies combining clinical phenotypes, environmental exposure, drug treatment, and genetic information should be conducted to evaluate the long-term predictive value of these SNPs for gynecological cancer risk in SLE patients and develop machine learning models based on multiomics data for personalized cancer risk assessments. Finally, high-density genotyping and sequencing of the MHC region should be conducted to clarify the specific role of functional variants in the SLE–uterine cancer association and explore new immunomodulatory strategies targeting related molecular pathways.

## 5. Conclusion

This study systematically revealed the significant genetic association between SLE and endometrial and uterine cancers through GWAS and cross-trait analysis, identifying several shared genetic variants, particularly rs2442719 and rs3131004 in the MHC region. These findings align with epidemiological observations of a protective effect of SLE on hormone-sensitive cancers, enhancing our understanding of the biological link between SLE and gynecological cancers. Our study emphasizes the dual nature of the relationship between SLE and cancer—both increasing the risk of certain cancers (e.g., NHL) and decreasing the risk of others (e.g., endometrial and uterine cancers). This complex pattern may reflect the dual role of the immune system in tumor surveillance: Enhanced immune surveillance may help clear some precancerous lesions, while chronic immune activation may increase the risk of DNA damage and lymphocyte proliferation. From a clinical and public health perspective, our findings provide scientific evidence for personalized cancer risk assessment and screening strategies for SLE patients. Although SLE patients may face an increased risk of some cancers, excessive concern may lead to unnecessary psychological burden and healthcare resource wastage. Physicians should develop balanced and precise cancer prevention and screening plans based on patients' genetic backgrounds, clinical features, and risk factors. Finally, our study provides genetic support for the concept of “beneficial autoimmunity” and opens up new perspectives for exploring the cross-regulation mechanisms between autoimmune diseases and tumor immunity. By deepening our understanding of these shared genetic and molecular mechanisms, innovative treatment strategies targeting both autoimmune diseases and cancers may be developed, offering new opportunities for comprehensive management of these two major diseases.

## Figures and Tables

**Table 1:** Information on the dataset for SLE and malignant neoplasm of the female reproductive system.

**Phenotypes**	**Year**	**Number of cases**	**Number of controls**	**Sample size**	**Race**
Systemic lupus erythematosus	2015	5201	9066	14,267	European population
Ovarian cancer	2021	779	246,729	247,508	European population
Vulvar cancer	2024	253	221,932	222,185	European population
Cervical cancer	2018	1934	245,606	247,540	European population
Endometrial cancer	2021	1477	246,063	247,540	European population
Vaginal cancer	2024	102	222,078	222,180	European population
Uterine body cancer	2024	3490	222,078	225,568	European population

**Table 2:** Multivariable analysis and sensitivity analysis.

**Outcome**	**Exposure**	**nsnp**	**Method**	**OR**	**95% CI**	**p** ** value**	**MVMR-cML (BIC_ ** **p** ** val)**	**Cochran's ** **Q**_**p**
Endometrial cancer	BMI	333	Inverse variance weighted	1.53	0.253, 0.599	< 0.001	3.04e−07	0.0025
			MR-Egger	2.19	0.256, 1.313	0.004		
			Median method	1.62	0.247, 0.714	< 0.001		
	Estradiol	333	Inverse variance weighted	0.55	−3.121, 1.936	0.646	6.30e−04	
			MR-Egger	0.50	−3.226, 1.831	0.589		
			Median method	0.95	−3.012, 2.912	0.974		
	SLE	308	Inverse variance weighted	0.96	−0.071, −0.008	0.014	1.91e−02	
			MR-Egger	0.96	−0.070, −0.007	0.017		
			Median method	1.00	−0.040, 0.049	0.844		
	CRP	333	Inverse variance weighted	0.99	−0.104, 0.082	0.814	6.37e−04	
			MR-Egger	0.99	−0.102, 0.084	0.851		
			Median method	1.02	−0.100, 0.132	0.788		

**Table 3:** Shared genetic associations of rs2442719 and rs3131004 in SLE and gynecological cancers.

**SNP**	**Chromosome position**	**Phenotype**	**Beta**	**SE**	**p**	**Effect direction**	**Heterogeneity ** **I** ^2^ ** (** **p** ** value)**
rs2442719	6: 31352661–31352861	SLE	−0.2231	0.0297	< 0.0001	—	
		Endometrial cancer	−0.0369	0.0179	< 0.05	—	
		Meta-analysis (fixed effects) (SLE–endometrial cancer)	−0.0866	0.0154	< 0.0001	—	96.5% (< 0.0001)
		Meta-analysis (random effects) (SLE–endometrial cancer)	−0.1285	0.0933	0.1675	—	
		Uterine cancer	−0.0583	0.0247	< 0.05	—	
		Meta-analysis (fixed effects) (SLE–uterine cancer)	−0.1254	0.0190	< 0.0001	—	94.5% (< 0.0001)
		Meta-analysis (random effects) (SLE–uterine cancer)	−0.1399	0.0824	0.0896	—	

rs3131004	6: 31127417–31127617	SLE	−0.1625	0.0295	< 0.0001	—	
		Endometrial cancer	−0.0474	0.0179	< 0.05	—	
		Meta-analysis (fixed effects) (SLE–endometrial cancer)	−0.0784	0.0153	< 0.0001	—	91.0% (0.0008)
		Meta-analysis (random effects) (SLE–endometrial cancer)	−0.1026	0.0575	0.0744	—	
		Uterine cancer	−0.0616	0.0249	< 0.05	—	
		Meta-analysis (fixed effects) (SLE–uterine cancer)	−0.1035	0.0190	< 0.0001	—	85.4% (0.0089)
		Meta-analysis (random effects) (SLE–uterine cancer)	−0.1108	0.0504	0.0280	—	

*Note:* All analyses were performed on SNPs located in the MHC region on Chromosome 6. Heterogeneity analysis was conducted using the Cochran's *Q* test, and the *I*^2^ value represents the proportion of variation in effect sizes attributable to heterogeneity between studies. Due to the observed high heterogeneity, the random effects model may provide more accurate effect estimates. All effect directions were consistent (negative), suggesting that these SNPs may have a protective effect on disease risk.

## Data Availability

The genetic data used in this study are from publicly accessible GWAS datasets. The data analyzed and obtained during this study can be found in the supporting information or in the data repositories provided in the original literature.

## Supporting Information

Supporting Information 1 Table S1: SNPs associated with SLE, cervical cancer, endometrial cancer, ovarian cancer, vulvar cancer, vaginal cancer, and uterine cancer. Descriptive analysis was performed to identify SNPs significantly associated with each cancer type. The SNPs were selected based on *p* < 5 × 10^−8^, and independent SNPs with *r*^2^ > 0.001 and kb = 10,000 were identified.

Supporting Information 2 Table S2: Shared genetic variants between SLE and uterine cancer, including 193 SNPs identified in the SLE and endometrial cancer analysis and 71 SNPs identified in the SLE and uterine cancer analysis. All SNPs were statistically significant (*p* < 0.05) and exhibited consistent effect directions in both diseases. Notably, rs2442719 and rs3131004 were the only SNPs identified in both comparisons.
