# Supplementary material for: Genetic Causal Relationship Between Systemic Lupus Erythematosus and Malignant Tumors of the Female Reproductive System: A GWAS Analysis in European Populations
Source: Hum Mutat. 2025 May 15;2025:7447886. doi: 10.1155/humu/7447886 (PMC12097853; doi:10.1155/humu/7447886)
Supplement: Supporting Information 1 — Table S1: SNPs associated with SLE, cervical cancer, endometrial cancer, ovarian cancer, vulvar cancer, vaginal cancer, and uterine cancer. Descriptive analysis was performed to identify SNPs significantly associated with each cancer type. The SNPs were selected based on p < 5 × 10^−8^, and independent SNPs with r2 > 0.001 and kb = 10,000 were identified. [file 7447886.f1.pdf]

endometrial cancer	SNP	beta.exposure	se.exposure	F_statistic	cervical cancer	SNP
1	rs10048743	0.231111721	0.041205628	31.45798263	1	rs10048743
2	rs10200680	-0.248461359	0.042483496	34.20402512	2	rs10200680
3	rs1078324	-0.713349888	0.078166469	83.28450457	3	rs1078324
4	rs10912578	0.246860078	0.030991799	63.44657025	4	rs10912578
5	rs1143679	0.58221562	0.039986634	212.0010492	5	rs1143679
6	rs12094036	-0.328504067	0.057859483	32.23535103	6	rs12094036
7	rs1270942	0.928219303	0.043382175	457.8029085	7	rs1270942
8	rs13019891	-0.562118918	0.029033597	374.8475828	8	rs13019891
9	rs13136219	-0.174353387	0.027786961	39.37120198	9	rs13136219
10	rs13332649	-0.314710745	0.037568252	70.17479705	10	rs13332649
11	rs143123127	0.470003629	0.084034201	31.28169463	11	rs143123127
12	rs143810596	-0.616186139	0.112573796	29.96051179	12	rs143810596
13	rs1464446	-0.328504067	0.040149728	66.94471337	13	rs1464446
14	rs150180633	0.928219303	0.068957329	181.1925467	14	rs150180633
15	rs17849501	0.810930216	0.049864233	264.4774686	15	rs17849501
16	rs2431697	-0.223143551	0.029296429	58.01486778	16	rs2431697
17	rs2459611	-0.261364764	0.045245001	33.36974369	17	rs2459611
18	rs2573219	0.587786665	0.042929166	187.4711983	18	rs2573219
19	rs268124	-0.186329578	0.032370264	33.1337902	19	rs268124
21	rs34703115	-0.616186139	0.104777606	34.58493046	21	rs34703115
22	rs35000415	0.587786665	0.041538955	200.2296207	22	rs35000415
23	rs35251378	-0.235722334	0.03242656	52.84448965	23	rs35251378
24	rs353608	-0.186329578	0.028019771	44.22159854	24	rs353608
25	rs3747093	0.262364264	0.034505488	57.81399936	25	rs3747093
26	rs4274624	0.559615788	0.032679116	293.2508752	26	rs4274624
27	rs4388254	0.378436436	0.060397671	39.25956682	27	rs4388254
28	rs4661543	-0.274436846	0.042375456	41.94266963	28	rs4661543
29	rs4916215	-0.223143551	0.033969323	43.15140062	29	rs4916215
30	rs58688157	-0.223143551	0.033564737	44.19795575	30	rs58688157
31	rs6671847	0.198850859	0.028965081	47.13086908	31	rs58721818
32	rs6679677	0.336472237	0.04648538	52.39208718	32	rs597808
33	rs6889239	0.277631737	0.031739962	76.51127084	33	rs6671847
34	rs7097397	-0.186329578	0.028711836	42.11547191	34	rs6679677
35	rs73050535	-0.713349888	0.124134163	33.02345849	35	rs6889239
36	rs73068668	-0.314710745	0.057490346	29.96631202	36	rs7097397
37	rs7768653	0.207014169	0.029689071	48.61910126	37	rs73050535
38	rs7823055	0.350656872	0.028620839	150.1066689	38	rs73068668
39	rs7899626	0.182321557	0.033253186	30.06142191	39	rs7768653
40	rs9852014	0.620576488	0.049272685	158.6273894	40	rs7823055
					41	rs7899626
					42	rs9852014

beta.exposure	se.exposure	F_statistic	ovarian cancer	SNP	beta.exposure	se.exposure
0.231111721	0.041205628	31.45798263	1	rs10048743	0.231111721	0.041205628
-0.248461359	0.042483496	34.20402512	2	rs10200680	-0.248461359	0.042483496
-0.713349888	0.078166469	83.28450457	3	rs1078324	-0.713349888	0.078166469
0.246860078	0.030991799	63.44657025	4	rs10912578	0.246860078	0.030991799
0.58221562	0.039986634	212.0010492	5	rs1143679	0.58221562	0.039986634
-0.328504067	0.057859483	32.23535103	6	rs12094036	-0.328504067	0.057859483
0.928219303	0.043382175	457.8029085	7	rs1270942	0.928219303	0.043382175
-0.562118918	0.029033597	374.8475828	8	rs13019891	-0.562118918	0.029033597
-0.174353387	0.027786961	39.37120198	9	rs13136219	-0.174353387	0.027786961
-0.314710745	0.037568252	70.17479705	10	rs13332649	-0.314710745	0.037568252
0.470003629	0.084034201	31.28169463	11	rs143123127	0.470003629	0.084034201
-0.616186139	0.112573796	29.96051179	12	rs143810596	-0.616186139	0.112573796
-0.328504067	0.040149728	66.94471337	13	rs1464446	-0.328504067	0.040149728
0.928219303	0.068957329	181.1925467	14	rs150180633	0.928219303	0.068957329
0.810930216	0.049864233	264.4774686	15	rs17849501	0.810930216	0.049864233
-0.223143551	0.029296429	58.01486778	16	rs2431697	-0.223143551	0.029296429
-0.261364764	0.045245001	33.36974369	17	rs2459611	-0.261364764	0.045245001
0.587786665	0.042929166	187.4711983	18	rs2573219	0.587786665	0.042929166
-0.186329578	0.032370264	33.1337902	19	rs268124	-0.186329578	0.032370264
-0.616186139	0.104777606	34.58493046	21	rs34703115	-0.616186139	0.104777606
0.587786665	0.041538955	200.2296207	22	rs35000415	0.587786665	0.041538955
-0.235722334	0.03242656	52.84448965	23	rs35251378	-0.235722334	0.03242656
-0.186329578	0.028019771	44.22159854	24	rs353608	-0.186329578	0.028019771
0.262364264	0.034505488	57.81399936	25	rs3747093	0.262364264	0.034505488
0.559615788	0.032679116	293.2508752	26	rs4274624	0.559615788	0.032679116
0.378436436	0.060397671	39.25956682	27	rs4388254	0.378436436	0.060397671
-0.274436846	0.042375456	41.94266963	28	rs4661543	-0.274436846	0.042375456
-0.223143551	0.033969323	43.15140062	29	rs4916215	-0.223143551	0.033969323
-0.223143551	0.033564737	44.19795575	30	rs58688157	-0.223143551	0.033564737
0.657520003	0.075594067	75.65585024	31	rs58721818	0.657520003	0.075594067
-0.162518929	0.029473642	30.40467007	32	rs597808	-0.162518929	0.029473642
0.198850859	0.028965081	47.13086908	33	rs6671847	0.198850859	0.028965081
0.336472237	0.04648538	52.39208718	34	rs6679677	0.336472237	0.04648538
0.277631737	0.031739962	76.51127084	35	rs6889239	0.277631737	0.031739962
-0.186329578	0.028711836	42.11547191	36	rs7097397	-0.186329578	0.028711836
-0.713349888	0.124134163	33.02345849	37	rs73050535	-0.713349888	0.124134163
-0.314710745	0.057490346	29.96631202	38	rs73068668	-0.314710745	0.057490346
0.207014169	0.029689071	48.61910126	39	rs7768653	0.207014169	0.029689071
0.350656872	0.028620839	150.1066689	40	rs7823055	0.350656872	0.028620839
0.182321557	0.033253186	30.06142191	41	rs7899626	0.182321557	0.033253186
0.620576488	0.049272685	158.6273894	42	rs9852014	0.620576488	0.049272685

F_statistic	vulvar cancer	SNP	beta.exposure	se.exposure	F_statistic	vaginal cancer
31.45798263	1	rs10048743	0.231111721	0.041205628	31.45798263	1
34.20402512	2	rs10200680	-0.248461359	0.042483496	34.20402512	2
83.28450457	3	rs1078324	-0.713349888	0.078166469	83.28450457	3
63.44657025	4	rs10912578	0.246860078	0.030991799	63.44657025	4
212.0010492	5	rs1143679	0.58221562	0.039986634	212.0010492	5
32.23535103	6	rs12094036	-0.328504067	0.057859483	32.23535103	6
457.8029085	7	rs1270942	0.928219303	0.043382175	457.8029085	7
374.8475828	8	rs13019891	-0.562118918	0.029033597	374.8475828	8
39.37120198	9	rs13136219	-0.174353387	0.027786961	39.37120198	9
70.17479705	10	rs13332649	-0.314710745	0.037568252	70.17479705	10
31.28169463	11	rs143810596	-0.616186139	0.112573796	29.96051179	11
29.96051179	12	rs1464446	-0.328504067	0.040149728	66.94471337	12
66.94471337	13	rs150180633	0.928219303	0.068957329	181.1925467	13
181.1925467	14	rs17849501	0.810930216	0.049864233	264.4774686	14
264.4774686	15	rs2431697	-0.223143551	0.029296429	58.01486778	15
58.01486778	16	rs2459611	-0.261364764	0.045245001	33.36974369	16
33.36974369	17	rs2573219	0.587786665	0.042929166	187.4711983	17
187.4711983	18	rs268124	-0.186329578	0.032370264	33.1337902	18
33.1337902	20	rs34703115	-0.616186139	0.104777606	34.58493046	20
34.58493046	21	rs35000415	0.587786665	0.041538955	200.2296207	21
200.2296207	22	rs35251378	-0.235722334	0.03242656	52.84448965	22
52.84448965	23	rs353608	-0.186329578	0.028019771	44.22159854	23
44.22159854	24	rs3747093	0.262364264	0.034505488	57.81399936	24
57.81399936	25	rs4274624	0.559615788	0.032679116	293.2508752	25
293.2508752	26	rs4388254	0.378436436	0.060397671	39.25956682	26
39.25956682	27	rs4661543	-0.274436846	0.042375456	41.94266963	27
41.94266963	28	rs4916215	-0.223143551	0.033969323	43.15140062	28
43.15140062	29	rs58688157	-0.223143551	0.033564737	44.19795575	29
44.19795575	30	rs58721818	0.657520003	0.075594067	75.65585024	30
75.65585024	31	rs597808	-0.162518929	0.029473642	30.40467007	31
30.40467007	32	rs6671847	0.198850859	0.028965081	47.13086908	32
47.13086908	33	rs6679677	0.336472237	0.04648538	52.39208718	33
52.39208718	34	rs6889239	0.277631737	0.031739962	76.51127084	34
76.51127084	35	rs7097397	-0.186329578	0.028711836	42.11547191	35
42.11547191	36	rs73050535	-0.713349888	0.124134163	33.02345849	36
33.02345849	37	rs73068668	-0.314710745	0.057490346	29.96631202	37
29.96631202	38	rs7768653	0.207014169	0.029689071	48.61910126	38
48.61910126	39	rs7823055	0.350656872	0.028620839	150.1066689	39
150.1066689	40	rs9852014	0.620576488	0.049272685	158.6273894	40
30.06142191						
158.6273894						

SNP	beta.exposure	se.exposure	F_statistic	uterine cancer	SNP	beta.exposure
rs10048743	0.231111721	0.041205628	31.45798263	1	rs10048743	0.231111721
rs10200680	-0.248461359	0.042483496	34.20402512	2	rs10200680	-0.248461359
rs1078324	-0.713349888	0.078166469	83.28450457	3	rs1078324	-0.713349888
rs10912578	0.246860078	0.030991799	63.44657025	4	rs10912578	0.246860078
rs1143679	0.58221562	0.039986634	212.0010492	5	rs1143679	0.58221562
rs12094036	-0.328504067	0.057859483	32.23535103	6	rs12094036	-0.328504067
rs1270942	0.928219303	0.043382175	457.8029085	7	rs1270942	0.928219303
rs13019891	-0.562118918	0.029033597	374.8475828	8	rs13019891	-0.562118918
rs13136219	-0.174353387	0.027786961	39.37120198	9	rs13136219	-0.174353387
rs13332649	-0.314710745	0.037568252	70.17479705	10	rs13332649	-0.314710745
rs143810596	-0.616186139	0.112573796	29.96051179	11	rs143810596	-0.616186139
rs1464446	-0.328504067	0.040149728	66.94471337	12	rs1464446	-0.328504067
rs150180633	0.928219303	0.068957329	181.1925467	13	rs150180633	0.928219303
rs17849501	0.810930216	0.049864233	264.4774686	14	rs17849501	0.810930216
rs2431697	-0.223143551	0.029296429	58.01486778	15	rs2431697	-0.223143551
rs2459611	-0.261364764	0.045245001	33.36974369	16	rs2459611	-0.261364764
rs2573219	0.587786665	0.042929166	187.4711983	17	rs2573219	0.587786665
rs268124	-0.186329578	0.032370264	33.1337902	18	rs268124	-0.186329578
rs34703115	-0.616186139	0.104777606	34.58493046	20	rs34703115	-0.616186139
rs35000415	0.587786665	0.041538955	200.2296207	21	rs35000415	0.587786665
rs35251378	-0.235722334	0.03242656	52.84448965	22	rs35251378	-0.235722334
rs353608	-0.186329578	0.028019771	44.22159854	23	rs353608	-0.186329578
rs3747093	0.262364264	0.034505488	57.81399936	24	rs3747093	0.262364264
rs4274624	0.559615788	0.032679116	293.2508752	25	rs4274624	0.559615788
rs4388254	0.378436436	0.060397671	39.25956682	26	rs4388254	0.378436436
rs4661543	-0.274436846	0.042375456	41.94266963	27	rs4661543	-0.274436846
rs4916215	-0.223143551	0.033969323	43.15140062	28	rs4916215	-0.223143551
rs58688157	-0.223143551	0.033564737	44.19795575	29	rs58688157	-0.223143551
rs58721818	0.657520003	0.075594067	75.65585024	30	rs58721818	0.657520003
rs597808	-0.162518929	0.029473642	30.40467007	31	rs597808	-0.162518929
rs6671847	0.198850859	0.028965081	47.13086908	32	rs6671847	0.198850859
rs6679677	0.336472237	0.04648538	52.39208718	33	rs6679677	0.336472237
rs6889239	0.277631737	0.031739962	76.51127084	34	rs6889239	0.277631737
rs7097397	-0.186329578	0.028711836	42.11547191	35	rs7097397	-0.186329578
rs73050535	-0.713349888	0.124134163	33.02345849	36	rs73050535	-0.713349888
rs73068668	-0.314710745	0.057490346	29.96631202	37	rs73068668	-0.314710745
rs7768653	0.207014169	0.029689071	48.61910126	38	rs7768653	0.207014169
rs7823055	0.350656872	0.028620839	150.1066689	39	rs7823055	0.350656872
rs9852014	0.620576488	0.049272685	158.6273894	40	rs9852014	0.620576488

se.exposure	F_statistic
0.041205628	31.45798263
0.042483496	34.20402512
0.078166469	83.28450457
0.030991799	63.44657025
0.039986634	212.0010492
0.057859483	32.23535103
0.043382175	457.8029085
0.029033597	374.8475828
0.027786961	39.37120198
0.037568252	70.17479705
0.112573796	29.96051179
0.040149728	66.94471337
0.068957329	181.1925467
0.049864233	264.4774686
0.029296429	58.01486778
0.045245001	33.36974369
0.042929166	187.4711983
0.032370264	33.1337902
0.104777606	34.58493046
0.041538955	200.2296207
0.03242656	52.84448965
0.028019771	44.22159854
0.034505488	57.81399936
0.032679116	293.2508752
0.060397671	39.25956682
0.042375456	41.94266963
0.033969323	43.15140062
0.033564737	44.19795575
0.075594067	75.65585024
0.029473642	30.40467007
0.028965081	47.13086908
0.04648538	52.39208718
0.031739962	76.51127084
0.028711836	42.11547191
0.124134163	33.02345849
0.057490346	29.96631202
0.029689071	48.61910126
0.028620839	150.1066689
0.049272685	158.6273894
