# Supplementary material for: Genetic Causal Relationship Between Systemic Lupus Erythematosus and Malignant Tumors of the Female Reproductive System: A GWAS Analysis in European Populations
Source: Hum Mutat. 2025 May 15;2025:7447886. doi: 10.1155/humu/7447886 (PMC12097853; doi:10.1155/humu/7447886)
Supplement: Supporting Information 2 — Table S2: Shared genetic variants between SLE and uterine cancer, including 193 SNPs identified in the SLE and endometrial cancer analysis and 71 SNPs identified in the SLE and uterine cancer analysis. All SNPs were statistically significant (p < 0.05) and exhibited consistent effect directions in both diseases. Notably, rs2442719 and rs3131004 were the only SNPs identified in both comparisons. [file 7447886.f2.pdf]

SNPs shared between SLE and Endometrial Cancer (n=193)	SNPs shared between SLE and Uterine Cancer (n=71)
rs10016018	rs10199181
rs10016325	rs10202642
rs10028805	rs1065043
rs10032160	rs1065044
rs1012899	rs1065048
rs10456362	rs1130422
rs1050458	rs113568276
rs10516483	rs1150668
rs10516486	rs1150690
rs10516487	rs115210925
rs1074706	rs11687659
rs1074707	rs1234317
rs10856963	rs12693582
rs11097757	rs13382604
rs11097760	rs1654774
rs11097761	rs17191234
rs111984068	rs2442719*
rs1125271	rs2647025
rs1131215	rs28366267
rs1131275	rs28366270
rs1131285	rs28366288
rs113299215	rs28366294
rs11736568	rs28366295
rs12163856	rs28366296
rs12331849	rs28366297
rs12498921	rs28366298
rs13106926	rs28366299
rs13107572	rs28366300
rs13107612	rs28366301
rs13108400	rs28366302
rs13128022	rs28366303
rs13135381	rs28366310
rs13136219	rs28366312
rs13136796	rs28366313

SNPs shared between SLE and Endometrial Cancer (n=193)	SNPs shared between SLE and Uterine Cancer (n=71)
rs13137133	rs28366314
rs13145909	rs28366315
rs13434472	rs28366316
rs141649669	rs28366317
rs1421627	rs28366319
rs144947706	rs28366330
rs147925578	rs28366334
rs1679709	rs28366335
rs1679732	rs28366337
rs169946	rs28366339
rs17200824	rs28366340
rs17266594	rs28366341
rs1778508	rs28366356
rs1778511	rs28366357
rs183352775	rs28366358
rs185819	rs28366359
rs187582489	rs28366360
rs1883259	rs28366362
rs1892250	rs28383312
rs1892251	rs28383313
rs1892252	rs2844623
rs1892253	rs28752509
rs1937126	rs28752510
rs1954598	rs3094221
rs1977199	rs3130561
rs1977200	rs3130565
rs1980453	rs3130629
rs2004640	rs3130980
rs200980	rs3131004*
rs2010291	rs4587163
rs2011465	rs502803
rs2011472	rs532098
rs2039069	rs535852
rs2052445	rs536810

SNPs shared between SLE and Endometrial Cancer (n=193)	SNPs shared between SLE and Uterine Cancer (n=71)
rs2074496	rs6434407
rs2080820	rs6434408
rs214049	
rs214050	
rs214066	
rs2216546	
rs2328887	
rs2328889	
rs2395475	
rs2395476	
rs241429	
rs2442719*	
rs2523572	
rs2523578	
rs2523579	
rs2523582	
rs2523585	
rs2523587	
rs2523589	
rs2523594	
rs2523595	
rs28412066	
rs2856448	
rs28625045	
rs28746784	
rs28787299	
rs28832872	
rs3131004*	
rs3132559	
rs34029191	
rs34749007	
rs35388091	
rs3792790	
rs3949215	

SNPs shared between SLE and Endometrial Cancer (n=193)	SNPs shared between SLE and Uterine Cancer (n=71)
rs3974649	
rs3974650	
rs4235402	
rs4236040	
rs4270588	
rs4270589	
rs4276281	
rs429150	
rs4296682	
rs4339214	
rs4365725	
rs4383809	
rs4426778	
rs4443287	
rs4463068	
rs4519797	
rs4554079	
rs4572884	
rs4576241	
rs4607219	
rs4610336	
rs4632883	
rs4637409	
rs4639076	
rs4643809	
rs4698839	
rs4698972	
rs4698974	
rs4711086	
rs4711090	
rs4712936	
rs4712969	
rs4712972	
rs4731530	

SNPs shared between SLE and Endometrial Cancer (n=193)	SNPs shared between SLE and Uterine Cancer (n=71)
rs4947311	
rs55768089	
rs6456685	
rs6456701	
rs6816787	
rs6850747	
rs6857428	
rs6900762	
rs6902211	
rs6903257	
rs6910549	
rs6932536	
rs6973520	
rs71536552	
rs71536564	
rs71537571	
rs71597109	
rs721803	
rs7386188	
rs7441177	
rs762502	
rs7656720	
rs7679882	
rs7682827	
rs7686702	
rs7698632	
rs7752195	
rs7774567	
rs7779671	
rs913455	
rs926326	
rs9266196	
rs9266197	
rs9266206	

SNPs shared between SLE and Endometrial Cancer (n=193)	SNPs shared between SLE and Uterine Cancer (n=71)
rs9266207	
rs9266217	
rs9266227	
rs9266230	
rs9266231	
rs9266232	
rs9266233	
rs9266234	
rs9266235	
rs9266239	
rs9266241	
rs9266242	
rs9266243	
rs9266244	
rs9266245	
rs9266250	
rs9266262	
rs9271744	
rs9295661	
rs932319	
rs936460	
rs9393654	
rs9999440	

Note: SNPs marked with an asterisk () appear in both comparison groups, suggesting they may play important roles in the genetic association between SLE and gynecological cancers.
